# Comparing a visual and verbal semantic memory test on the effects of gender, age and education as assessed in a cognitively healthy sample: A pilot study in the development of the international neuropsychological test profile—a tablet-based cognitive assessment

**DOI:** 10.1007/s40211-020-00355-9

**Published:** 2020-06-30

**Authors:** Theresa Heidinger, Johann Lehrner

**Affiliations:** Department of Neurology, Medical University of Vienna, Währinger Gürtel 18–20, 1097 Vienna, Austria

**Keywords:** Semantic memory, Incidental learning, Tablet testing, Dementia, Semantisches Gedächtnis, Inzidentelles Lernen, Tabletbbasierter Test, Demenz

## Abstract

**Background:**

Due to the increase of dementia diagnoses and individuals interested in monitoring their cognitive status, practical new neuropsychological tests are needed. Tablet-based tests offer a good alternative to traditional paper–pencil tests, as they can be completed remotely and independently. This study assessed two semantic memory tests (verbal and visual memory), in the scope of the creation of a new tablet-based battery—the International Neurocognitive Profil (INCP)—on the influences of demographic variables.

**Methods:**

In all, 46 cognitively healthy participants who recruited at the memory clinic of the Medical University of Vienna were included in this study. They were asked to complete two tests of semantic memory and implicit learning: Capital Knowledge (CK) using verbal input and Flag Knowledge (FK) using visual input. Performance on the two tests was analysed according to influences of gender and age using two analyses of variance. Post hoc comparisons between age and gender groups were performed. In addition, correlational analyses were computed to assess strengths of association with age, gender and education.

**Results:**

FK- and CK-based measures were found to be influenced by demographic variables with semantic memory measures being significantly influenced by gender and education while incidental memory measures were influenced by age. Performances differed between FK and CK showing that the mode of testing (visual, verbal) affected participant’s performance.

**Conclusion:**

These findings are important for the creation of normative samples for both CK and FK. Furthermore, this study underlines the importance of using different testing modes when assessing individuals’ semantic memory.

## Abstract (de)

**Hintergrund:**

Aufgrund der Zunahme von Demenzdiagnosen und der steigenden Anzahl von Personen, die daran interessiert sind, ihren kognitiven Status zu kennen, sind neue, praktische neuropsychologische Testverfahren erforderlich. Tabletbasierte Tests bieten eine gute Alternative zu Paper-Pencel-Verfahren. In dieser Studie wurden zwei semantische Gedächtnistests (verbales und visuelles Gedächtnis) im Rahmen der Erstellung einer neuen tabletbasierten Testbatterie, dem International Neurocognitive Profil (INCP), in Bezug auf die Einflüsse demographischer Variablen untersucht.

**Methodik:**

Insgesamt wurden 46 kognitiv gesunde Teilnehmer in diese Studie eingeschlossen, die an der Gedächtnisklinik der Medizinischen Universität Wien rekrutiert worden waren. Sie wurden gebeten, zwei Tests zum semantischen Gedächtnis und zum zufälligen Lernen zu absolvieren: Capital Knowledge (CK) mit verbalem Input und Flag Knowledge (FK) mit visuellem Input. Die Leistung in beiden Tests wurde nach Einflüssen von Geschlecht und Alter mithilfe von zwei Varianzanalysen analysiert. Es wurden Post- hoc-Vergleiche zwischen Alters- und Geschlechtsgruppen durchgeführt. Darüber hinaus wurden Korrelationsanalysen berechnet, um die Stärke der Assoziation mit Alter, Geschlecht und Bildung zu bewerten.

**Ergebnisse:**

Es wurde festgestellt, dass FK- und CK-basierte Messwerte von demographischen Variablen beeinflusst werden, wobei Geschlecht und Bildung einen signifikanten Einfluss auf die Messwerte des semantischen Gedächtnisses haben. Das inzidentelle Gedächtnis hingegen wird vom Alter beeinflusst. Die Gedächtnisleistungen zwischen FK und CK waren unterschiedlich und zeigten, dass der Testmodus (visuell, verbal) Einfluss auf die Leistung der Teilnehmer hatte.

**Schlussfolgerung:**

Diese Ergebnisse sind wichtig für die Erstellung normativer Stichproben sowohl für die CK als auch für die FK. Darüber hinaus unterstreicht diese Studie die Bedeutung der Verwendung verschiedener Testmodi bei der Beurteilung des semantischen Gedächtnisses.

## Background

For years the number of people diagnosed with dementia has been steadily increasing in Austria. According to the Austrian Alzheimer’s Association the number of people suffering from dementia is around 100,000 with the projected numbers for 2050 being more than twice as many [1]. This is said to be due to the aging population in this country [2] as the incidence and prevalence of dementia increase with age [1]. This real increase in dementia patients is accompanied by an increase of the number of people fearful of developing dementia in part due to the heightened public awareness of the disease. A great number of people attending memory clinics present with perceived cognitive problems, despite unimpaired performance on cognitive tests which is termed subjective cognitive decline (SCD). Previous research has demonstrated, however, that SCD may indicate the first symptomatic manifestation of Alzheimer’s dementia (AD) [3].

Due to the incline in dementia patients and the increase of people interested in monitoring their cognitive state, the need for efficient and easy tools to help distinguish normal cognitive status from cognitive impairment in patients is more pressing than ever. Due to time and financial restrictions on medical professionals as well as restrictions on patients such as feasibility of travel to clinics and medical practices and a possibility for bias in testing due to an unfamiliar testing environment, a self-administered, home-based testing solution would be the most effective way to screen a large number of potential patients.

Over the past decades, computerized neurocognitive tests have gained popularity for assessing dementia and cognitive decline [4]. Overall, computerized tests are said to be superior to traditional paper–pencil testing in that they offer precise standardization of administration [4, 5]. They are also more time and cost efficient than paper–pencil tests and can be used in large-scale testing more easily [5]. With tablet-based tests, technological innovation has been further integrated into neuropsychological testing. Tablets may be advantageous to computers as touch screens have been argued to be more intuitive for older adults than computers using a mouse or a keyboard [6]. They also allow more mobility in testing and may improve engagement and therefore patient compliance [6]. One study evaluating the feasibility of the Computerized Cognitive Composite for Preclinical Alzheimer’s Disease (C3-PAD)—a home-based tablet test done by healthy elderly over the course of a week (multiple measurements)—demonstrated feasibility of this type of assessment, with authors reporting high reliability and validity of cognitive data recorded in a home environment as compared with in-clinic assessment [7].

The background of this current study is the development of a proprietary tablet-based neurocognitive battery—the International Neurocognitive Profile (INTP)—that should be useful to assess cognitive impairment remotely and to differentiate SCD and mild cognitive impairment (MCI) cases. While current cognitive assessments for the detection of preclinical dementia use composites of different cognitive domains specifically sensitive for AD pathology, this new battery should test all six neurocognitive domains as proposed in the Diagnostic and Statistical Manual of Mental Disorders V (i.e. language, learning and memory, executive function, complex attention, social cognition and perceptual–motor function) [8]. This should lead to a more detailed depiction of impairment/resources and could therefore provide information on preclinical levels of other dementia types aside from the common AD, which would set this battery apart from other batteries currently available [9, 10]. Another goal for battery development is to produce a repeatable measure useful for monitoring cognitive development over time, which is important for detecting and determining the progression of impairment. This could improve our understanding of the progression from the preclinical to the clinical stage of cognitive impairment. As a first step in the development of the INTP, subtests need to be newly developed and psychometrically tested. Thus, in this pilot study, two novel tests assessing verbal and visual semantic memory and implicit learning that were completed by a cognitively healthy sample were examined regarding the effects of participant characteristics such as gender, age and education.

Semantic memory is responsible for the individual’s ability to acquire and maintain general knowledge about the world [11]. It encapsulated all memory on facts, concepts, words, associations and meanings that were not encoded with episodic associations [12]. Semantic memory, unlike episodic memory does not show impairment in healthy aging [11–13] but is impaired in dementia [14]. In fact, one study reported it to be one of the first functions to be impaired in AD patients with retrospective analyses determining a lower semantic memory task score in future AD developed than the reference group 12 years prior to diagnosis [15]. Semantic memory measures have been reported to differentiate between levels of cognitive impairment. Aside from countless studies, describing the significant differences found on measures of faces [16, 17], supportive data has been reported for measures using buildings and world capitals differentiating between healthy controls, MCI and AD [17]. In a healthy sample, semantic memory should be unaffected by age due to this function remaining relatively stable over the life span (as compared to episodic memory) [11, 13]. It should relate to education with more highly educated people reportedly performing better on semantic memory tasks [13, 18]. Concerning gender, previous research was divided with some papers stating an advantage for female participants [19], while others reported equality of performance between genders [11, 13] and one reported a male advantage in semantic memory measures [20].

Implicit learning is characterized by a lack of awareness during the acquisition of knowledge [21]. It is the counterpart of explicit or intentional learning, wherein the person is actively and consciously gathering knowledge. In terms of neuropsychological testing, implicit learning can be tested by not informing participants on later memory evaluation [22]. Likewise semantic memory reports regarding gender influences on implicit learning show inconsistent results with some researchers reporting a slight advantage of women over men [23]. Conversely, a recent paper suggested that gender does not show any effect on incidental memory [22]. Age has been shown to affect incidental learning, with previous research finding a clear advantage of younger versus older participants [22]. The impact of education on implicit learning has not been addressed in the scientific literature. As intelligence, which is positively correlated with education level [24], has been found to lack an association with implicit learning, it is plausible that education also does not show any association or effect on implicit learning.

Because a visual and verbal memory test were used to assess both semantic memory and incidental learning, this study aims to compare the results from these two tests. Due to the lack of comparative studies, this study is therefore using a more exploratory approach to compare these measures. Focussing on the effects of demographic variables on semantic memory and incidental learning, based upon previous research, this study aimed at testing the following hypotheses: (1) Gender differences may be seen in semantic memory and incidental learning. Due to inconsistent findings, no hypothesis on the existence or direction of effect of gender can be made. (2) Age is expected to impact incidental learning with younger participants performing better than older participants. Age is not expected to affect semantic memory. (3) Education is expected to affect semantic memory with more highly educated individuals outperforming less educated participants, but not incidental learning.

## Methods

### Sample and procedure

This study used a sample of healthy participants who were recruited from individuals accompanying patients to the memory clinic at the Medical University of Vienna. In addition, students working at the clinic at the time of development were included as subjects. Participants were asked to provide written consent prior to taking part in the study. As the specification for sample was cognitively fit individuals, the sole exclusion criterion was a score of 8 or below on the Vienna Visuo-Constructive Test (VVT 3.0) copy task as this score has been shown to be indicative of possible neurological impairment [25]. The VVT 3.0 is a test of the individual’s visuo-constructional abilities. In the immediate copy task, participants were asked to copy three objects (analogue clock set at 11:10, two intercepting pentagons and a three-dimensional cube) as accurately as possible. After a delay, the participant was asked to draw the three items from memory (delayed recall task). Both the copy and the delayed recall task were scored broadly and in detail. This study used only the broad score (0–10) on the immediate copy task for participant evaluation. The used cut-off was set at 9, excluding participants scoring 8 or below, which had been found to successfully exclude patients with MCI or AD [25].

All participants were asked to complete the VVT 3.0 (immediate copy and delayed recall) and the INTP. This preliminary version of the INTP consisted of tests assessing memory, language, executive function (calculation) based on tests sourced from www.psimistri.com, as well as some questionnaires on mood, subjective memory impairments and olfaction. Procedure consisted of signing of the consent form, doing the VVT 3.0 copy task, the INTP, and the finally the VVT 3.0 delayed recall task. Total testing time was about thirty minutes. Apart from one free recall tests, all tests on the INTP, including the two tests analysed in this study, were completed independently, with a test administrator standing by in case of questions. Demographic information as well as test scores were recorded on the INTP interface.

### Measures

#### Capital Knowledge and Flag Knowledge of the INTP

For this study two tests of the INTP were analysed: Capital Knowledge (CK) and Flag Knowledge (FK). Both tests were novel tests of semantic memory, using verbal (CK) and visual (FK) input to test semantic memory. In both tasks, participants were asked to match an item (capital name or country flag) to the correct country which was presented with a distractor country below the item on the screen (e.g. Kuala Lumpur—Japan, Malaysia). If the participant did not know the correct answer, they were encouraged to guess. Participants were presented with 20 forced choice items. Afterwards they were shown the same 20 items with new distractor items in a random order twice over (three presentation rounds overall). Participants’ choice was recorded (correct/incorrect). Due to the repeated presentation of the same 20 items participants were expected to perform better in rounds 2 and 3 than round 1, solely because they had previously matched the country names/flags. This round 2 and round 3 performance was interpreted as informing on participants incidental learning. Semantic memory was defined as their success rate in the first round of presentation with a higher number of correct answers implying a stronger semantic memory. Participant’s implicit learning was defined as the performance rate in round 2 and 3. Due to the novelty of these tasks, no psychometric information can be reported on these measures.

#### Demographic measures

Participants’ gender, age (in years) and education level (schooling in number of years) were recorded and used for analysis. In addition, the sample was divided into groups ‘young’ and ‘old’ according to the median.

### Statistical analysis

Analyses were performed using SPSS software (version 25, IBM, Armonk, NY, USA). Depending on variable distribution, parametric or nonparametric tests were used to evaluate the data. Two mixed model analysis of variance were computed to assess the effects of gender and age on all three rounds of performance (i.e. semantic memory and incidental learning measures). Subsequently, post hoc tests were computed to assess group differences on significant variables (t-tests, U‑tests). Finally, correlational analyses were included to assess the association of all three descriptive variables and measures of semantic memory and incidental learning of both tests. Inferential statistics was interpreted at an alpha level of α = 0.05, in accordance with scientific practice. Correlational coefficients and effect sizes were evaluated according to Cohen’s guidelines [26].

## Results

### Descriptive statistics

#### Sample characteristics

**Fig. 1:**
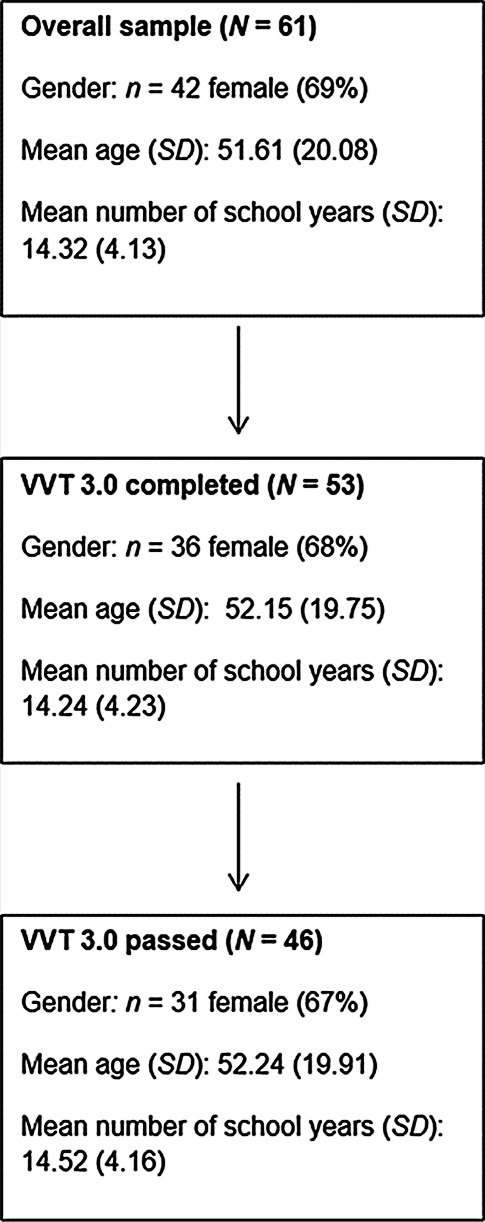
Participant flow chart showing descriptive characteristics of the sample over stages of exclusion. *VVT* *3.0* Vienna Visuo-constructional Test [25], *N* sample size, *n* subsample size, *SD* standard deviation

The sample which originally included 61 participants was restricted to participants which had completed the VVT 3.0 and had scored a minimum of 9 points on the immediate copy task. Therefore, the sample used for statistical analysis included 46 participants (67% female). Despite the exclusion of participants, mean age, mean number of school years attended and the gender distribution remained similar to the original sample. A participant flow chart of the sample restriction is presented in Fig. 1. Participants mean age was 52 years with a standard deviation of almost 20 years. This large deviation is due to the inclusion of both a younger group (students) and an older group (accompanying persons) into the sample. Participants were aged from 21 years to 82 years (*median* = 53 years). For comparative analysis, participants were classed as ‘young’ or ‘old’ by the median. Each category included 23 participants with a similar age rage: younger = 21–52 years, older = 54–82 years. Gender distribution was very similar between groups with *n* = 8 male and *n* = 15 female participants in the younger category, and *n* = 7 male and *n* = 16 female participants in the older category. Mean education level was 14.52 (4.16) years of education with number of school years ranging from 8 to 23 years. Overall, the sample was well educated. Due to the extensive span of this variable and the small sample size, neither the effect of education on semantic memory or incidental memory nor differences according to education level (years of education) were computed. However, correlational analyses of education and these measures were computed.

### Inferential statistics

**Fig. 2:**
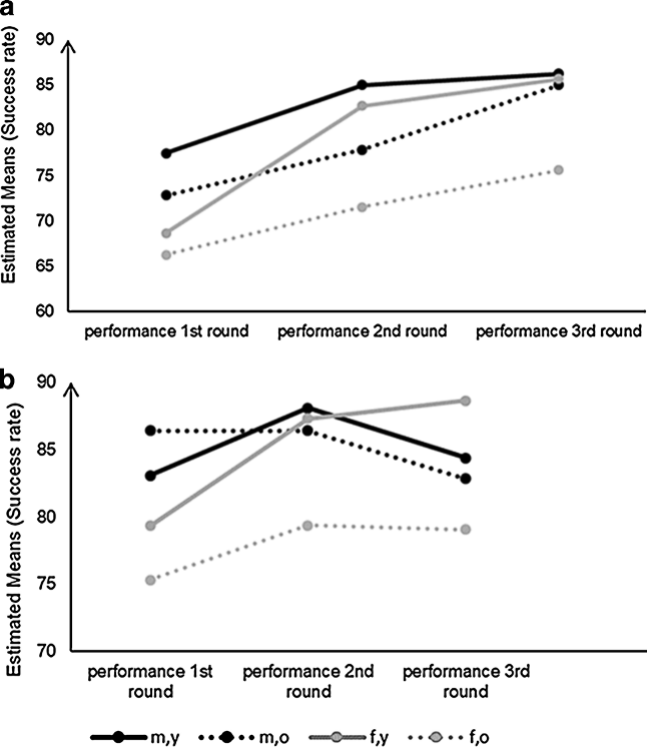
Estimated means for (**a**) Capital Knowledge (CK) and (**b**) Flag Knowledge (FK) performances across three rounds by group. *m,y* male, young, *m,o* male, old, *f,y* female, young, *f, o* female, old

To analyse the impact of gender and age on performance over all three rounds of presentation in CK and FK mixed-model analyses of variance were computed using performance (rounds 1, 2, 3) as the within subject factor and gender and age group as between subject factors. For computations using CK data all assumptions of the mixed-model analysis of variance (ANOVA) were fulfilled. A significant main effect of performance was found (*F* (2,84) = 18.68, *p* < 0.001, partial *η*^*2*^ = 0.31) which can be interpreted as performance differences being prominent in the population, irrespective of age or gender group. Also, statistically significant main effects of gender (*F* (1,42) = 8.19, *p* < 0.01, partial *η*^*2*^ = 0.15) and age (*F* (1,42) = 8.19*, p* < 0.0, partial *η*^2^ = 0.16) were found. None of the tested interaction effects were found to be statistically significant. Fig. 2 shows the estimated marginal means of performance per group (male, young; male, old; female, young; female, old) over the three time points in CK.

For computations using FK data, equality of covariance and sphericity could be assumed based on the dataset. Even though the normality of residuals could not be confirmed in this dataset, ANOVA was still performed, as previous research has reported the mixed ANOVA to be robust to nonnormal data [27]. As a result, none of the main effects nor the interaction effects were shown to be significant. Performance could not be assumed to significantly differ between time points. Neither significant gender differences nor age differences could be reported based on this sample. The estimated marginal means of performance of all groups (male, young; male, old; female, young; female, young) over the three rounds of presentation in FK are also shown in Fig. 2.

#### Post hoc tests: analysing differences

To illustrate the differences found in this sample, descriptive information on performance data (semantic memory and incidental learning) according to gender and age groups is provided in Table 1. Differences were further explored post hoc for CK data, as the ANOVA using FK data showed no significant effects. After checking the distribution of data, t‑tests were computed. For gender, semantic memory differences were found to be significant (*t* (44) = 2.42, *p* = 0.02) with a moderate to strong effect (*d* = 0.77). On incidental learning, gender differences did not reach significance (second round:* t* (44) = 1.33, *p* = 0.19, *d* = 0.41, third round: *t* (44) = 1.73, *p* = 0.09, *d* = 0.57). While no significant differences due to age group were found on semantic memory (*t* (44) = 1.37, *p* = 0.18, *d* = 0.40), incidental learning measures were found to be significantly influenced by age group (second round: *t* (44) = 3.27, *p* < 0.01, *d* = 0.97, third round: *t* (44) = 2.75, *p* < 0.01, *d* = 0.81). Age group had a strong effect on incidental learning measures. These results suggest that the verbal semantic memory test CK is influenced by gender and age, with gender influencing semantic memory and age influencing the incidental learning.

**Table 1:** Descriptive performance scores over all three rounds and according to gender and age group

Test		*n*		1st round performance (semantic memory)	2nd round performance (implicit learning)	3rd round performance (implicit learning)
				*M (SD)*	*M (SD)*	*M (SD)*
Capital Knowledge	Overall	46	–	69.24 (12.52)	78.48 (11.44)	82.17 (9.76)
	Differentiated by gender	31	Male	75.33 (11.10)	81.67 (11.90)	85.67(7.99)
		15	Female	66.29 (12.25)	76.94 (11.08)	80.48 (10.19)
	Differentiated by age group	23	Young	71.74 (11.83)	83.48(10.16)	85.87(9.00)
		23	Old	66.74(12.93)	73.48(10.60)	78.48(9.22)
Flag Knowledge	Overall	46	–	79.67 (10.92)	84.57 (10.90)	83.70(10.35)
	Differentiated by gender	31	Male	84.67(8.12)	87.33(8.63)	83.67(9.35)
		15	Female	77.26(11.39)	83.23(11.73)	83.71(10.95)
	Differentiated by age group	23	Young	80.65(9.69)	87.61(9.64)	87.17(8.90)
		23	Old	78.70(12.18)	81.52(11.43)	80.22(10.71)

*n* Sample size, *M* mean, *SD* standard deviation

#### Correlational analysis

As a last step, correlational analyses were computed to identify associations between semantic memory and incidental learning measures (performance round 1; performance scores 2 and 3) of both CK and FK and participant variables gender, age and education. Using the Shapiro Wilk test, the normality of variables was assessed. In CK all variables apart from age were found to be normally distributed, while age as well as all performance measures (semantic memory and incidental memory) were found to be nonnormally distributed in FK. For CK, Pearson’s correlation was computed on performance × education, Spearman’s correlation on performance × age and point-biserial correlation on performance × gender. As performance measures and the age variable were found to be nonnormally distributed in FK, Spearman’s correlations were used for all but the gender × performance correlations (point-biserial correlation). Correlational coefficients are provided in Table 2.

**Table 2:** Correlations

	Gender (r_pb_)	Age (r_s_)	Education (r/r_s_)
Capital Knowledge			
Semantic memory (performance 1st round)	−0.34*	−0.16	0.38*
Incidental learning (performance 2nd round)	−0.25	−0.41**	0.25
Incidental learning (performance 3rd round)	−0.30	−0.37*	0.30
Flag Knowledge			
Semantic memory (performance 1st round)	−0.32*	−0.05	0.31*
Incidental learning (performance 2nd round)	−0.18	−0.39**	0.30*
Incidental learning (performance 3rd round)	<0.01	−0.47**	0.23

*r*_*pb*_ point-biserial correlation; *r*_*s*_ Spearman’s correlation; *r* Pearson’s correlation

*significant at a level of 0.05, **significant at a level of 0.01

Gender correlated significantly with semantic memory in CK and FK. In both tests moderate negative associations were found with gender, with women performing significantly worse than men. Gender was not significantly associated with incidental learning. Age showed a significant relation to incidental learning in both CK and FK while it did not significantly relate to semantic memory in either CK or FK. In both tests moderate significant negative correlation coefficients were found for second and third round performance and age indicating that younger participants showed better incidental learning than older participants. Education was shown to be significantly related to semantic memory in both CK and FK with participants who reported higher education outperforming less educated individuals. Correlations were positive and of moderate strength. Apart from FK second-round performance, which also correlated significantly with education, all other correlations using incidental learning measures did not reach significance.

## Practical conclusion

This study aimed at comparing two tablet-based tests of verbal (CK) and visual (FK) semantic memory on the impacts of descriptive variables gender, age and education. Although CK and FK were assumed to measure the same cognitive functions (semantic memory, incidental learning) this study found differences between these tests. Overall, CK seemed to have been more difficult than FK with participants showing lower performance scores in all rounds on CK compared to FK (Table 1). Using repeated measure ANOVA (analysis of variance) it was shown that CK was influenced significantly by gender and age. Both variables were assessed with post hoc tests and were found to affect the test in the hypothesized ways. As the same model using FK data failed to show significant effects of any variable this study cannot speak to the influences of gender or age on FK over and above correlational analysis results. Possible explanations for the lack of significance of the effects on the ANOVA could be an inappropriate sample due to the small size or participants’ general competence level on this test.

Gender was found to influence CK semantic knowledge, with men outperforming women. This finding was peculiar but not unexpected, as the scientific literature had been divided on the influence of gender on semantic memory [11, 13, 19]. It was interesting that women scored lower in both CK and FK semantic memory measures in this sample with additional correlational analysis revealing significant relationships of gender and first round performances on both tests. Understanding this male superiority was challenging—one possible explanation could have been a better knowledge level of men in the field of geography. In this study gender did not influence incidental learning measures in CK or FK. This could be interpreted as incidental learning being independent of gender.

Hypotheses regarding age were confirmed in this study. In CK, age group was found to significantly impact incidental learning measures but not the semantic memory measure. Due to previous findings on semantic memory remaining stable in healthy aging [11, 13], this finding was expected. Incidental learning was impacted by age group with younger participants ‘out-learning’ older participants. This finding was also consistent with the scientific literature [22]. Association with incidental learning measures but not with semantic memory measures were also found in correlative analysis using both CK and FK data. Overall, age did seem to impact semantic memory tests, which may be due to differences in learning abilities rather than semantic memory changes with age.

Education was only assessed using correlational analysis due to the broad range of education levels and the small sample size. In this study, education was found to relate to semantic memory measures of CK and FK which concurred with the posed hypothesis. It was also found to have no relationship with incidental learning on CK but did have a relationship with the second-round performance in FK. However, because FK data were skewed and the analysis of variance did not result in any significant effects, this finding should be treated with caution. Overall education was shown to relate to semantic memory and, in most computations, did not relate to incidental learning. This result is suggestive of education level affecting semantic memory tests.

Findings from this study need to be considered when creating normative data for CK and FK as norms must be adapted for gender, age and education. Overall CK and FK resulted in different performance data with this healthy sample performing better on FK than CK in all three rounds, which may hint at that test being too easy. FK may therefore be the preferable test to CK for a clinical population, as it may better distinguish the cognitively fit from the cognitively impaired. However, a recent study, advocated the use of both verbal and visual tests of semantic memory in the assessment of (amnestic) cognitive impairment [28]. The authors stated that in using only verbal tests, which did find the majority (60%) of amnestic MCI patients, 27% of patients who showed only impairment in visual tests were overlooked. This was especially problematic, since patients showing impairments on visual memory tests were also more likely to have multidomain aMCI and were had a higher rate of progression to AD [28]. Showing differing results between CK and FK in a healthy sample may justify the inclusion of both tests in the future tablet- based test.

In conclusion, this study showed differential outcomes of two novel tests of semantic memory in a healthy sample of participants, therefore hinting at a benefit of keeping both tests in future INTP versions. It also evidenced that the demographic variables of gender, age and education affected participant performance on these tests. It further demonstrated that effects were due to variables affecting either semantic memory storage or incidental learning. Overall, all three variables were shown to affect CK and FK and should therefore be considered in the creation of normative data for these tests.
